# *Ancrocorticia populi *gen. nov., sp. nov, isolated from the symptomatic bark of *Populus *× *euramericana* canker

**DOI:** 10.1002/mbo3.792

**Published:** 2019-01-17

**Authors:** Guan‐tang Xu, Han Xue, Chun‐gen Piao, Min‐wei Guo, Yong Li

**Affiliations:** ^1^ The Key Laboratory of State Forestry Administration on Forest Protection, Research Institute of Forest Ecology Environment and Protection Chinese Academy of Forestry Beijing China

**Keywords:** Ancrocorticia populi gen. nov., sp. Nov., canker, comparative genome, percentage of conserved proteins, polar lipids

## Abstract

A Gram‐staining positive facultative anaerobic, non‐motile strain, sk1b4^T^, was isolated from canker of symptomatic bark tissue of a *Populus* × *euramericana*. 16S rRNA gene sequence analyses showed that strain sk1b4^T^ shared the highest similarity with *Arcanobacterium phocisimile *(94.1%). Within the phylogenetic tree, the novel isolate formed a distinct branch from *Actinobaculum*, *Arcanobacterium*, and *Trueperella*. The percentage of conserved proteins calculated from genomic sequence indicated a low level of relatedness between the novel strain and its phylogenetic neighbors. Growth of the novel strain occurred at temperatures between 10 and 41°C, and within a pH range of 6.0–9.0; optimal growth occurred at 30°C and at pH 6.0–9.0. Growth also occurred within a NaCl concentration of 1%–5% (w/v). The major fatty acids of the strain were C_14:0_, C_16:0_, and C_18:1_ *ω*9*c*_, _and major polar lipids were glycolipid, phosphatidylinositol mannoside, phospholipid, diphosphatidylglycerol, and phosphatidylglycerol. Respiratory quinone was absent. On the basis of phenotypic and genotypic characteristics, we propose that the novel isolate should be classified as a novel species in a new genus: *Ancrocorticia populi *gen. nov., sp. nov. The type strain is sk1b4^T^ (=CFCC 14564^T^= KCTC 39919^T^).

## 1 INTRODUCTION

The *Actinomycetaceae* created by Buchanan in 1918 are a family of bacteria in the order *Actinomycetales* that contains the medically important genus *Actinomyces *(Buchanan, 1918). The family was originally used to accommodate many diverse organisms such as members of the genera *Actinobacillus*, *Leptotrichia*, *Actinomyces,* and *Nocardia*. Following several revisions, membership of the family was restricted to bacterial species. Recently, the application of modern taxonomic techniques such as chemotaxonomics, numerical phonetics, and molecular genetics to the respective organisms has resulted in inclusion of the genera *Actinobaculum*, *Actinomyces*, *Arcanobacterium*, *Flaviflexus*, *Mobiluncus*, *Trueperella*, *Varibaculum*, and *Actinotignum *in the *Actinomycetaceae *(Schaal, Yassin, & Stackebr, 2006; Yassin et al., 2015).

Strain sk1b4^T^ was isolated from the symptomatic bark of *Populus *×* euramericana *with canker disease using standard dilution plating techniques during our bacterial diversity investigation in Puyang City, Henan Province, China. This study is directed toward the taxonomic investigation of the isolate and describes its characterization using a polyphasic approach.

## 2 MATERIALS AND METHODS

### 2.1 Phylogenetic analyses

Genomic DNA was extracted using a bacterial genomic DNA extraction kit (Biomed company, China). The 16S rRNA gene was then amplified and sequenced using universal primers 8F (5‐AGAGTTTGATCCTGGCTCAG‐3) and 1525R (5‐AAGGAGGTGATCCAGCC‐3) (Baker, Smith, & Cowan, 2003; Lane, 1991). A 1,459‐bp sequence of the novel strain was obtained and compared with other species for sequence similarity using EzTaxon‐e online software (https://www.ezbiocloud.net/identify) (Yoon et al., 2017). Phylogenetic analysis was performed according to the method of Fang et al. (2016). In brief, the phylogenetic tree based on the 16S rRNA gene sequence was constructed using MEGA 5.0 by the neighbor‐joining, maximum‐likelihood, and maximum‐parsimony methods (Tamura et al., 2011). The resulting tree was evaluated using 1,000 boostrap replicates.

### 2.2 Genome sequencing and percentage of conserved proteins (POCP) analysis

Genome sequencing was performed as described by Li, Song, Guo, Wang, and Liang (2016). In brief, the library was constructed by 300‐ to 500‐bp DNA fragmentation using the TruSeq™ DNA Sample Prep Kit. Sequence assembly was performed using the SOAPdenovo assembly method v2.04 (http://soap.genomics.org.cn/). Coding sequences were predicted using Glimmer 3.02 software (http://www.cbcb.umd.edu/software/glimmer/). Functional annotation of the protein sequences was performed against the nonredundant GenBank database using BLASTP. RNA genes, that is, rRNAs, tRNAs, and other RNAs, were predicted using the RNAmmer and ARAGORN algorithms (Lagesen et al., 2007; Laslett & Canback, 2004). POCP analysis between a pair of genomes was performed according to Qin et al. (2014). It was calculated as [(C1+C2)/(T1+T2)] 100%, where C1 and C2 represent the numbers of conserved proteins in the two genomes being compared, respectively, and T1 and T2 represent the total numbers of predicted proteins in the two genomes being compared. For a pair of genomes, each genome was used as the query genome to perform the BLASTP search and the proteins matched with an *E*‐value of <1e^−5^.

### 2.3 Chemotaxonomic analyses

For the analyses of chemotaxonomic markers, the strain was grown aerobically in tryptic soy broth (TSB; Difco) at 30°C for 72 hr and was harvested at the end of the logarithmic period. Isoprenoid quinones were extracted from strain sk1b4^T^ cells as described by Collins, Pirouz, Goodfellow, and Minnikin (1977), analyzed by high‐performance liquid chromatography (Du et al., 2013; Groth et al., 1997), and results were confirmed by liquid chromatography‐mass spectrometry. Polar lipids of strain sk1b4^T^ were investigated by two‐dimensional thin‐layer chromatography, as reported by Minnikin et al. (1984). Cell wall peptidoglycans were isolated and analyzed by the methods of Schleifer and Kandler (1972), Schleifer (1985), MacKenzie (1987), and Groth, Schumann, Weiss, Martin, and Rainey (1996). Cell wall sugars were analyzed as described by Staneck and Roberts (1974). For cellular fatty acid analysis, the novel strain was cultured in tryptic soy agar (Difco) at 30°C, and cells were harvested after 72 hr. The fatty acid methyl ester was saponified, methylated, extracted, and quantified according to the standard protocol of the Sherlock Microbial Identification System (MIDI, version 6.0) (Sasser, 2001).

### 2.4 Morphological observation and physiological tests

Gram straining was performed according to Jenkins, Richard, and Daigger (1986), and oxidase activities were tested as described by Smibert and Krieg (1994). Catalase activities were measured using 3% H_2_O_2_, while cell and flagella morphology were determined by transmission electron microscopy. Phase‐contrast microscopy (TMS‐F; Nikon) was used to determine the motility of the strain. Physiological growth parameters were carried out as recommended by Fang et al. (2016), and the pH range for bacterial growth was determined according to Gomeri (1955) using the following buffers: 0.1 M citric acid/sodium citrate (pH 4.0–6.0), 0.2 M Na_2_HPO_4_/NaH_2_PO_4_ (pH 6.0–8.0), 0.1 M Na_2_CO_3_/NaHCO_3_ (pH 8.0–9.5), and 0.1 M Na_2_HPO_4_/NaOH (pH 10.0–11.0). Tolerance of 1%, 3%, 5%, 8%, and 9% (w/v) NaCl was tested in TSB (pH 7.0). The effects of different growth temperatures for sk1b4^T ^were assessed in TSB with incubation at 10, 37, 41, and 45°C. The sk1b4^T^ was grown under aerobic condition for tolerance tests.

### 2.5 Biochemical analyses

Enzymic activity, carbon source utilization, and other physiological and biochemical tests were performed using API 20E, API ZYM, and API 20NE systems according to the manufacturer's instructions. The results were read after 48 hr incubation at 30°C. The Biolog GN2 microplate was also used to determine the carbon source utilization ability of the novel and reference species. Detailed results are shown in Table 1 and species description.

**Table 1:** Differential characteristics between the strain sk1b4^T^ and related genera of the family *Actinomycetaceae*

Characteristic	1	2	3	4
Catalase	Negative	Negative	Negative	Positive
G + C content (mol%)	58.22%	55%–57%	50%–60%	50%–57%
Poplar lipid	DPG, PG, PI, PIM, GL	DPG, PG, PI, PIM	DPG, PG, PI, AbGL, GL	DPG, PG, PI
Respiratory quinones	Absent	MK‐10(H4)	Absent	MK‐9(H4)
Major fatty acid	C_14:0_, C_16:0_, C_18:1_ *ω*9c	C_16:0_, C_18:1 _ *ω*9c, C_14:0_, C_18:0_	C_16:0_, C_18:1 _ *ω*9c, C_14:0_, C_18:0_	C_16:0_, C_18:1 _ *ω*9c, C_18:0_, C_18:2 _ *ω*6,9c/anteiso‐C_18:0_
Cell wall sugars	Glucose, rhamnose	Glucose, rhamnose,	Glucose, rhamnose, deoxytalose	Glucose, rhamnose
Catalase cell morphology	Straight curved rods	Straight to slightly curved rods	straight to slightly curved rods	Slender, irregular rods or granular, segmented cocci
O_2_ metabolism	F	AN/F	AN/F	F
Temperature	10–41°C	30–43°C	*n*	4–42°C
Optimal temperature	30°C	37°C	*n*	37°C
Hydrolysis of Esculin	+	−	−	−
Nitrate reduction	+	−	+	−

Notes

−: negative; +: positive; A: aerobic; AbGL: choline‐containing phosphoglycolipid; AN: anaerobic; DPG: diphosphatidylglycerol; F: facultatively anaerobic; GL: glycolipid; *n*: not mentioned; PG: phosphatidylglycerol; PI: phosphatidylinositol; PIM: phosphatidylinositol mannoside; PL: phospholipid.

MK‐*n*(Hx) means a partially hydrogenated menaquinone with × hydrogen atoms on the side chain containing *n* isoprene units.

Genera: 1, sk1b4^T^; 2, *Actinobaculum *(data from Lawson, Falsen, Akervall, Vandamme, & Collins, 1997); 3, *Actinotignum *(data from Yassin et al. (2015)); 4, *Arcanobacterium *[data from Collin, Jones, and Schofield (1982), Yassin, Hupfer, Siering, and Schumann (2011) and Hijazin et al. (2012)].

## 3 RESULTS

### 3.1 Phylogenetic analyses

**Figure 1:**
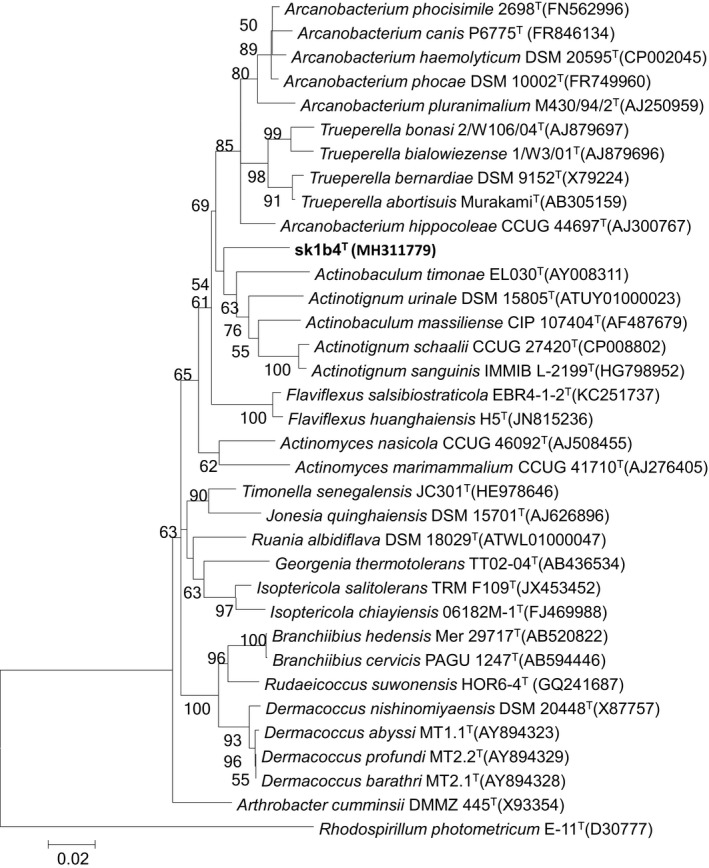
Neighbor‐joining phylogenetic tree based on the almost complete 16s rRNA gene, showing the relationships between the novel strain and reference species. The tree was constructed using MEGA 5. Bootstrap values >50% (based on 1,000 resamplings) are shown. The scale bar corresponds to 0.02 substitutions per nucleotide position

According to 16S rRNA sequence analysis, strain sk1b4^T^ shared most similarity with *Arcanobacterium phocisimile *(94.1%), followed by *Arcanobacterium haemolyticum *(94.0%). It shared less than 94% sequence similarity with all other validly published names. Within the phylogenetic tree, the novel isolate formed a distinct branch from *Actinobaculum*, *Arcanobacterium*, and *Trueperella*, indicating that it should be classed as a novel species in a novel genus within the family *Actinomycetaceae* (Figure 1, Supporting Information Figure S1).

### 3.2 Genome sequencing and POCP analysis

The genome of the novel isolate is 2,651,737 bp long (GenBank accession number: QETB00000000), and the final assembly identified eight large contigs (>500 bp). The maximum sequence length is 1,080,388 bp, and the G + C content is 58.77%. In total, 2,388 genes are predicted, including 2,289 protein‐coding genes and 46 RNAs. The detailed genome information of strain sk1b4^T^ and related reference are shown in Supporting Information Table S1. They have similar genome sizes and gene numbers, while strain sk1b4^T ^has the largest genome size and gene numbers of all six genome sequences. A POCP of 50% would be a proper genomic parameter for delimiting the prokaryotic genus boundary according to Qin *et al* (2014). The extremely low POCP value compared with other members of the family *Actinomycetaceae* support sk1b4^T^ as a novel genus. Intergenera POCP analyses between sk1b4^T^ and strains from five genera of the *Actinomycetaceae* had values ranging from 41% to 47%, which indicating that strain sk1b4^T^ does not belong to any validly described genera from this family (Table 2).

**Table 2:** POCP values for pairs of genomes between strain sk1b4^T^ and other five genera in the family *Actinomycetaceae*

Strains name	sk1b4^T^	C1	C2	T1	T2	POCP, %
*Actinobaculum suis *DSM 20639	sk1b4^T^	925	955	1,799	2,368	45
*Actinotignum urinale *DSM 15805	sk1b4^T^	850	868	1,700	2,368	42
*Actinobaculum massiliense* FC3	sk1b4^T^	952	973	1,783	2,368	46
*Trueperella pyogenes* TP6375	sk1b4^T^	805	829	1,661	2,368	41
*Arcanobacterium haemolyticum* DSM 20595	sk1b4^T^	995	1,001	1,857	2,368	47

### 3.3 Chemotaxonomic analyses

Isoprenoid quinones were shown to be absent, while the polar lipid profile included glycolipid (GLs), phosphatidylinositol mannoside (PIM), phospholipid (PL), diphosphatidylglycerol (DPG), phosphatidylglycerol (PG), two unidentified phosphatidylinositols, two unidentified PLs, an unidentified aminolipid (AL) and five unidentified lipids. The DPG, PG, and PI are present in sk1b4^T^ and relatives. However, the presence of AL is one an important characteristic used to distinguish strain sk1b4^T^ and related reference genera. The main peptidoglycans of the isolate cell wall were lysine, alanine, and glutamic acid, and the main whole‐cell sugar was glucose and rhamnose. The main cellular fatty acids (>10%) were C_14:0_ (41.3%) and C_16:0_ (27.2%); C_10:0_ (3.3%), C_12:0_ (3.4%), C_16:1_ *ω*9*c* (7.9%), C_18:1_ *ω*9*c* (9.9%), and C_18:0_ (4.9%) were also detected. The amount of C_14:0 _can be used to differentiate the novel strain from reference genera (*Actinobaculum*, *Actinotignum*, *Arcanobacterium*). Detailed differentiating characteristics of the novel species and reference genera are shown in Supporting Information Table S2.

### 3.4 Morphological observation and physiological tests

Cells are Gram‐staining positive, facultative anaerobic, oxidase‐negative, non‐motile, and catalase‐negative. The strain can grow from 10 to 41°C and at pH 6.0–9.0, with optimal growth at 30°C and pH 8.0–9.0. Growth occurred in the presence of 1%–5% (w/v) NaCl. Cells are 1 µm long and 0.5 µm wide. The esculin ferric citrate can be hydrolyzed.

### 3.5 Biochemical analyses

The cell is positive for the activities (API ZYM) of esterase (C4), esterase lipase (C8), leucine arylamidase, valine arylamidase, cystine arylamidase, acid phosphatase, *β*‐galactosidase, *α*‐glucosidase, and *β*‐glucosidase, and negative for the activities of alkaline phosphatase, naphthol‐AS‐BI‐phosphohydrolase, lipase (C14), *α*‐galactosidase, trypsin, *α*‐chymotrypsin, *β*‐glucuronidase, *α*‐mannosidase, and *α*‐fucosidase. Other results are listed in the species description and Table 1.

## 4 DISCUSSION

Previous studies showed that a sequence identity of 94.5% or below for two 16S rRNA genes is strong evidence for distinct genera (Yarza et al., 2008, 2014). In the present study, the novel strain shared <94.5% identity with 16S rRNA gene sequences of all published species in the *Actinomycetaceae*. Phylogenetic analysis based on 16S rRNA gene sequences showed that sk1b4^T^ was not closely related to any genera within the family, suggesting that it should belong to a separate genus. Moreover, the POCP values between the novel strain and its closely related genera neighbors less than prokaryotic genus boundary (50%), further suggesting that sk1b4^T^ should be present a novel species of a novel genus in the family *Actinomycetaceae*.

The absence of respiratory quinones from the novel strain can also be used to differentiate it from genera *Actinobaculum* and *Arcanobacterium*, while the presence of PIM and absence of choline‐containing phosphoglycolipid AbGL in the polar lipids distinguishes it from *Actinotignum. *The O_2_ metabolism, catalase activity, cell morphology, and fatty acid composition were also useful to distinguish the novel isolate from reference genera (Table 1). Based on these analyses, we suggest that sk1b4^T^ should be assigned to a novel species of a novel genus in the family *Actinomycetaceae*, and propose the name *Ancrocorticia populi* gen. nov., sp. nov.

### 4.1 Description of *Ancrocorticia* gen. nov.

#### 4.1.1 *Ancrocorticia* (An.cro.cor.ti.cia. L. gen. n. *corticis* of bark)

Cells are Gram‐staining positive, facultative anaerobic, oxidase‐negative, non‐motile, and catalase‐negative. The polar lipid profile includes GL, PIM, PL, DPG, PG, two unidentified phosphatidylinositols, two unidentified PLs, an unidentified aminolipid, five unknown lipids, and moderate amounts of three unidentified GLs. Menaquinones are absent. The main fatty acids are C_14:0_ and C_16:0_. The type species is *Ancrocorticia populi.*

### 4.2 Description of *Ancrocorticia populi* sp. nov.

#### 4.2.1 *Ancrocorticia populi* (po'pu.li. L. fem. gen. n. *populi *of the poplar tree)

Negative for the activities of l‐tryptophane, d‐glucose, l‐arginine, urea, gelatin, l‐arabinose, d‐mannose, d‐mannitol, *N*‐acetyl‐glucosamine, d‐maltose, potassium gluconate, capric acid, adipic acid, malic acid, trisodium citrate, and phenylacetic acid, and positive for nitrophenyl‐*β‐* d‐galactopyranoside (API 20NE). Acid can be produced from d‐mannitol, d‐sucrose, l‐arabinose, d‐melibiose, and amygdalin, but not from 2‐nitrophenyl‐*β‐* d‐galactopyranoside, l‐arginine, l‐lysine, l‐ornithine, trisodium citrate, sodium thiosulfate, l‐tryptophane, sodium pyruvate, inositol, d‐sorbitol and l‐rhamnose (API 20E). The strain is positive for d‐galactonic acid lactone, d‐galacturonic acid, l‐proline, and l‐arabinose, but negative for i‐erythritol, d‐melibiose, acetic acid, P‐hydroxyphenylacetic acid, bromosuccinic acid, l‐histidine, urocanic acid, *α*‐cyclodextrin, d‐fructose, *β*‐methyl d‐glucoside, cis‐aconitic acid, itaconic acid, succinamic acid, hydroxy l‐proline, inosine, dextrin, l‐fucose, d‐psicose, citric acid, *α*‐ketobutyric acid, glucuronamide, l‐leucine, uridine, glycogen, d‐galactose, d‐raffinose, formic acid, *α*‐ketoglutaric acid, alaninamide, l‐ornithine, thymidine, Tween‐40, gentiobiose, l‐rhamnose, *α*‐ketovaleric acid, d‐alanine, l‐phenylalanine, phenylethylamine, Tween‐80, *α*‐d‐glucose, d‐sorbitol, d,l‐lactic acid, l‐alanine, putrescine, *N*‐acetyl‐d‐galactosamine, *m*‐inositol, sucrose, d‐gluconic acid, malonic acid, l‐alanyl‐glycine, l‐pyroglutamic acid, 2‐amino ethanol, *N*‐acetyl‐d‐glucosamine, *α*‐d‐lactose, d‐trehalose, d‐glucosaminic acid, propionic acid, l‐asparagine, d‐serine, 2,3‐butanediol, adonitol, lactose, turanose, d‐glucuronic acid, quinic acid, l‐aspartic acid, l‐serine, glycerol, maltose, xylitol, *α*‐hydroxybutyric acid, d‐saccharic acid, l‐glutamic acid, l‐threonine, d, l‐*α*‐glycerol, d‐arabitol, d‐cellobiose, d‐mannitol, d‐mannose, methyl pyruvate, mono‐methyl succinate, *β*‐hydroxybutyric acid, *γ*‐hydroxybutytric acid, sebacic acid, succinic acid, glycyl‐L‐aspartic acid, glycyl‐l‐glutamic acid, d,l‐camitine, *γ*‐amino butyric acid, glucose‐1‐phosphate, and glucose‐6‐phosphate. Polar lipid profiles include GL, PIM, PL, DPG, PG, two unidentified phosphatidylinositols, two unidentified PLs, an unidentified aminolipid, five unidentified lipids, and moderate amounts of three unidentified GLs. Isoprenoid quinones are absent. The main cellular fatty acids are C_14:0_ and C_16:0_. The DNA G + C content is 58.77 mol%. The type strain is sk1b4^T^ (=CFCC 14656^T^= KCTC 39919^T^), and the strain was isolated from the canker of *Populus* × *euramericana*.

## CONFLICT OF INTEREST

The authors declare that there are no conflicts of interest.

## AUTHORS CONTRIBUTION

Chun‐gen Piao, Min‐wei Guo, and Yong Li conceived and designed the experiments. Guan‐tang Xu and Han Xue wrote the manuscript.

## ETHICS STATEMENT

None required.

## Data Availability

Data of the genome and 16S rRNA gene sequences of sk1b4^T^ are available on GenBank website, the accession number are QETB00000000 and MH311779, respectively. Supporting data are available in supplementary file.
