# Supplementary material for: Ancrocorticia populi gen. nov., sp. nov, isolated from the symptomatic bark of Populus × euramericana canker
Source: Microbiologyopen. 2019 Jan 17;8(7):e00792. doi: 10.1002/mbo3.792 (PMC6612551; doi:10.1002/mbo3.792)

Ancrocorticia populi gen. nov., sp. Nov, isolated from the symptomatic bark of _Populus_ × _euramericana_ canker

Guan-tang Xu，Xue Han, Chun-gen Piao, Lai-fa Wang, Min-wei Guo, Yong Li

[1]The Key Laboratory of State Forestry Administration on Forest Protection, Research Institute of Forest Ecology Environment and Protection, Chinese Academy of Forestry, Beijing 100091, China;

[2]Puyang Academy of Forestry, Puyang 457000, China.

Supplementary Fig. S1 The ML phylogenetic tree based on the complete 16s rRNA gene, showing the relationships between the novel strain and reference species. The tree was constructed using MEGA5 (Tamura *et al*. 2011). Bootstrap values > 50 % (based on 1,000 resamplings) are shown. The scale bar corresponds to 0.05 substitutions per nucleotide position.

Supplementary Fig. S2. Transmission electron micrograph of a cell of strain sk1b4^T after incubation for 72h at 30 °C on TSA medium. Bar, 1 µm.

Supplementary Fig. S3. Polar lipids profile of the novel strain separated by two-dimensional thin-layer chromatography. They were detected by spraying with a molybdatophosphoric acid reagent glycolipid (GL), phosphatidylinositol mannoside (PIM), phospholipid (PL), diphosphatidylglycerol (DPG) and phosphatidylglycerol (PG).

Supplementary Table S1. The genome information of novel strain and reference strains.

Name	RefSeq	Size (Mb)	GC%	Protein	rRNA	tRNA	Other RNA	Gene
sk1b4^T	QETB00000000	2.65	58.8	2289	3	47	3	2388
Actinobaculum suis DSM 20639	NZ_FNAU00000000	2.2	57.8	1,723	6	45	3	1,777
Actinobaculum massiliense FC3	NZ_CYUL00000000	2.07	60.2	1,701	13	47	3	1,790
Actinotignum urinale DSM 15805	NZ_ATUY00000000	1.92	50.9	1,572	5	45	3	1,691
Trueperella pyogenes TP6375	NZ_CP007519	2.34	59.5	2,037	6	46	3	2,124
Arcanobacterium haemolyticum DSM 20595	NC_014218	1.99	53.1	1,750	12	50	3	1,858

Supplementary Table S2. Cellular fatty acid composition(%) between the strain sk1b4^T and related genera of the family *Actinomycetaceae*

Genera: 1, sk1b4^T; 2, *Actinobaculum* (data from Lawson et al. (1997)); 3, *Actinotignum* (data from Yassin et al. (2015)); 4, *Arcanobacterium* (data from Collin et al. (1982), Yassin et al. (2011) and Hijazin et al. (2011))

Sum Feature 3: $C_{16:1}$ ω7c/$C_{16:1}$ ω6c; Sum Feature 5: $C_{18:2}$ ω6,9c; Sum Feature 8: $C_{18:1}$ ω7c; “-”: did not detected the fatty acid.

Fatty acid (%)	1	2	3	4
$C_{10:0}$	3.3	1.0---1.5	-	-
$C_{12:0}$	3.4	1.8---2.0	-	-
$C_{14:0}$	41.1	7.7---19.0	-	0.7---3.0
$C_{16:1}$ ω9c	7.3	1.1	-	0.6---3.0
$C_{16:0}$	27.3	22.9---41.5	10.7-22.3	22.5---43.0
$C_{18:1}$ ω9c	9.9	24.2---34.8	50.7-73.0	23.1---37.0
$C_{18:0}$	4.9	7.0---14.4	4.1-13.3	15.1---28.1
Sum Feature 3	1.3	-	-	-
Sum Feature 5	-	6.6	<1	1.1---2.0
$C_{16:1}$ ω7c	-	3.3	-	0.7---2.8
Sum Feature 8	1.5	-	-	-

Fig. S1

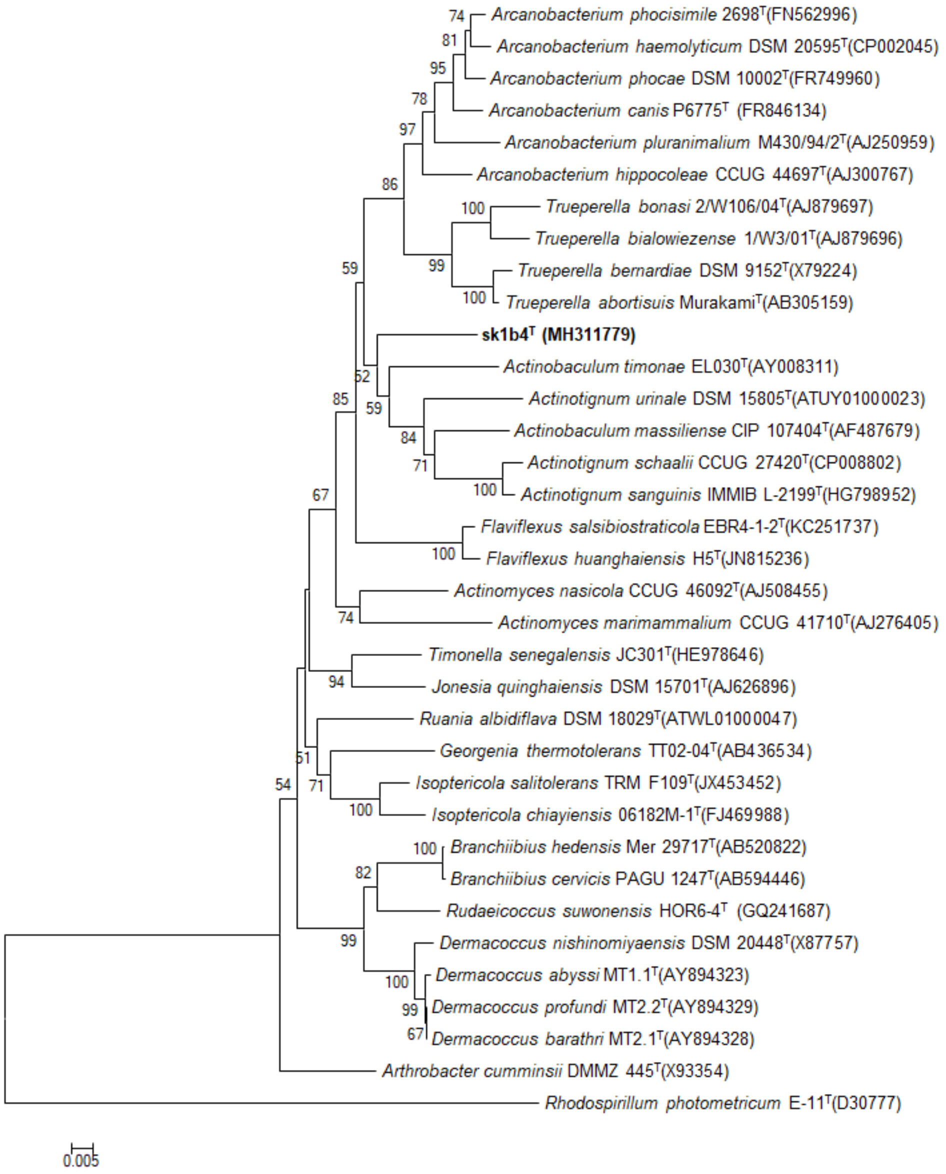
Arcanobacterium phocisimile 2698^T(FN562996)
Arcanobacterium haemolyticum DSM 20595^T(CP002045)
Arcanobacterium phocae DSM 10002^T(FR749960)
Arcanobacterium canis P6775^T (FR846134)
Arcanobacterium pluranimalium M430/94/2^T(AJ250959)
Arcanobacterium hippocoleae CCUG 44697^T(AJ300767)
Trueperella bonasi 2/W106/04^T(AJ879697)
Trueperella bialowiezense 1/W3/01^T(AJ879696)
Trueperella bernardiae DSM 9152^T(X79224)
Trueperella abortisuis Murakami^T(AB305159)
sk1b4^T (MH311779)
Actinobaculum timonae EL030^T(AY008311)
Actinotignum urinale DSM 15805^T(ATUY01000023)
Actinobaculum massiliense CIP 107404^T(AF487679)
Actinotignum schaalii CCUG 27420^T(CP008802)
Actinotignum sanguinis IMMIB L-2199^T(HG798952)
Flaviflexus salsibiostraticola EBR4-1-2^T(KC251737)
Flaviflexus huanghaiensis H5^T(JN815236)
Actinomyces nasicola CCUG 46092^T(AJ508455)
Actinomyces marimammalium CCUG 41710^T(AJ276405)
Timonella senegalensis JC301^T(HE978646)
Jonesia quinghaiensis DSM 15701^T(AJ626896)
Ruania albidiflava DSM 18029^T(ATWL01000047)
Georgenia thermotolerans TT02-04^T(AB436534)
Isoptericola salitolerans TRM F109^T(JX453452)
Isoptericola chiayiensis 06182M-1^T(FJ469988)
Branchiibius hedensis Mer 29717^T(AB520822)
Branchiibius cervicis PAGU 1247^T(AB594446)
Rudaeicoccus suwonensis HOR6-4^T (GQ241687)
Dermacoccus nishinomiyaensis DSM 20448^T(X87757)
Dermacoccus abyssi MT1.1^T(AY894323)
Dermacoccus profundi MT2.2^T(AY894329)
Dermacoccus barathri MT2.1^T(AY894328)
Arthrobacter cumminsii DMMZ 445^T(X93354)
Rhodospirillum photometricum E-11^T(D30777)
0.005

Fig. S2

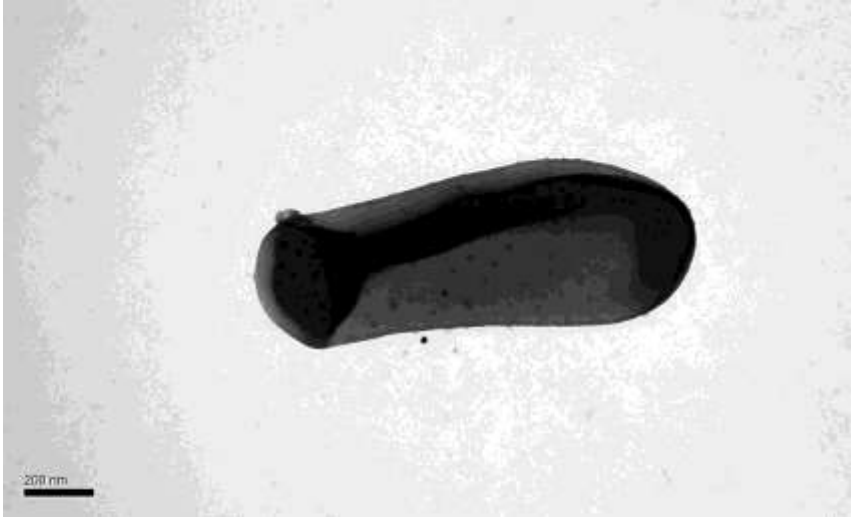

Fig. S3

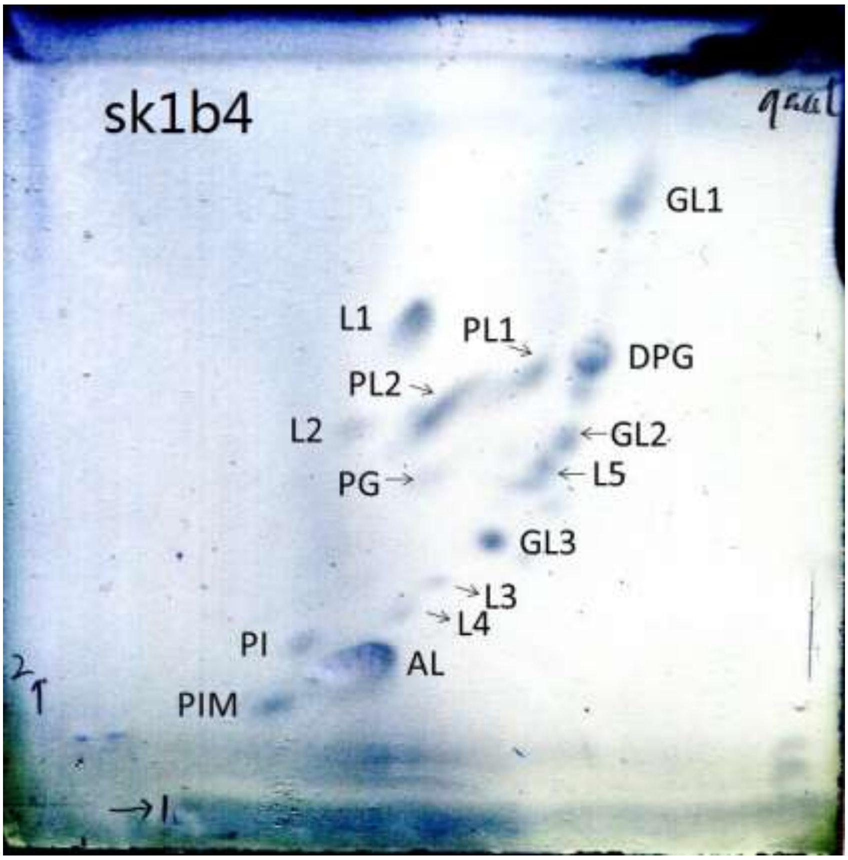

Supplement: Supplementary file 1 [file MBO3-8-e00792-s001.pdf]
